# Giant cutaneous horn in an African woman: a case report

**DOI:** 10.1186/1752-1947-1-170

**Published:** 2007-12-05

**Authors:** Peter M Nthumba

**Affiliations:** 1 Department of Surgery, AIC Kijabe Hospital, PO Box 20 Kijabe 00220, Kenya, Africa

## Abstract

**Introduction:**

A cutaneous horn is a conical projection of hyperkeratotic epidermis. Though grossly resembling an animal horn, it lacks a bony core. These lesions have been well described in Caucasian patients, as well as in a number of Arabic and Asian patients.

**Case presentation:**

A young female presented with a large 'horn' of five-year duration, arising from a burn scar. Excision and scalp reconstruction were performed. Histology was reported as verrucoid epidermal hyperplasia with cutaneous horn.

**Conclusion:**

This may be the first documentation of this lesion in a black African. Although likely rare, it should be considered in the differential diagnosis of dermatologic lesions. Up to 40% of cutaneous horns occur as part of a premalignant or malignant lesion, and surgical extirpation with histological examination is thus more important than the curiosity surrounding these lesions.

## Introduction

A cutaneous horn, or cornu cutaneum, is a dense hyperkeratotic conical projection of skin arising from an unusual cohesiveness of keratinized material. It resembles an animal horn grossly, but lacks a bony core, histologically consisting of concentric layers of cornified epithelial cells. Most have a yellow-white color, and may be straight or curved and twisted, and vary from a few millimeters to several centimeters in length [1,2]. Cutaneous horns may arise from any part of the body, and only 30% arise from the face and scalp. They are thought to result from underlying benign, premalignant or malignant pathology, in 61.1%, 23.2% and 15.7% of cases respectively [3].

## Case presentation

**Figure 1:**
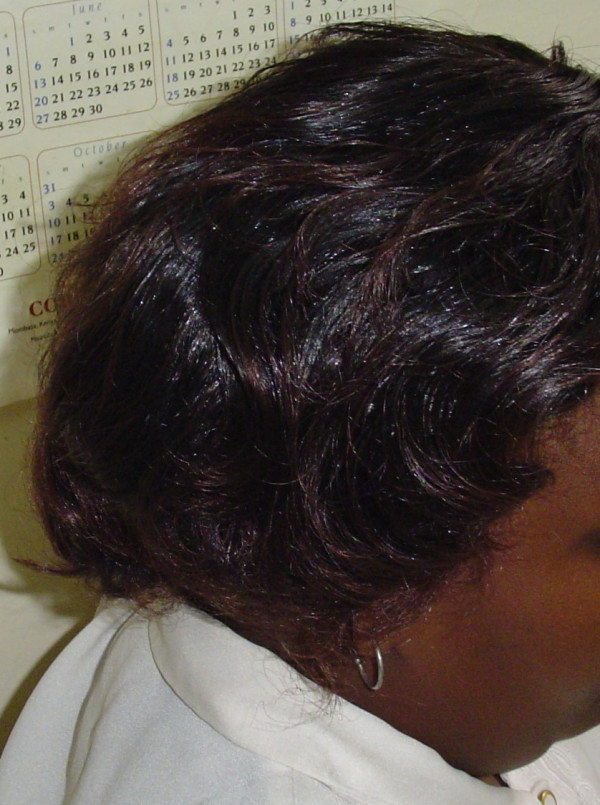
Patient wearing a wig to hide the cutaneous horn.

A 28 year old female patient presented to AIC Kijabe Hospital (KH) with a large 'horn' growing from her right parietal region. She had suffered a thermal burn of this same area at the age of 5 years. She had successfully concealed the scalp burn scar using a wig all her life (Figure 1), until three years prior to presentation, when she noticed a mass developing on the scar. The mass gradually increased in size, making it more and more difficult to conceal. It also began bleeding spontaneously, but with no associated pain.

**Figure 2:**
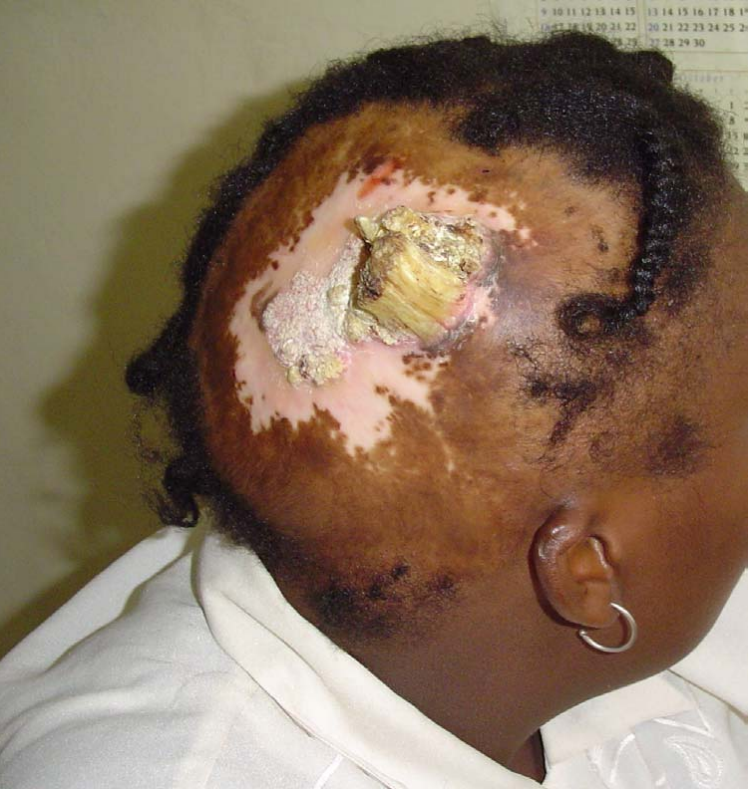
Cutaneous Horn. Note area of vitiligo and old burn scar surrounding the horn.

**Figure 3:**
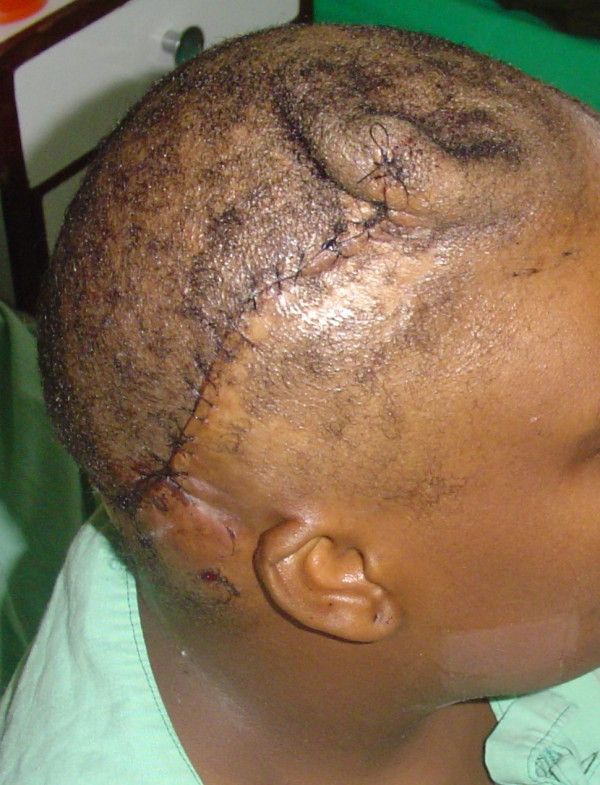
After initial skin grafting and placement of a tissue expander, the entire area of alopecia was excised, and reconstruction was performed with expanded scalp.

On examination, she had a large 15 cm × 15 cm area of alopecia over the right temporo-parietal region of her scalp. A golden-yellow colored horn with a base diameter of 3 cm and a height of 6 cm sat in the middle of the scar, with an extension of a mass of similar consistency posteriorly, measuring 5 cm by 4 cm. An area of hypopigmentation encircled this mass (Figure 2). The lesion was excised, and the defect covered with a skin graft (Figure 3). A tissue expander was inserted into the adjacent scalp to enable scalp expansion and reconstruction (Figure 3).

Histology was reported as verrucoid epidermal hyperplasia with a cutaneous horn, and vitiligo. There was no evidence of malignancy.

The patient was satisfied with the scalp reconstruction especially as it allowed her to do away with the use of a wig.

## Discussion

A sizeable number of people with cutaneous horns have been reported in the medical literature, almost entirely among Caucasians from Europe [1-4], with a few additional reports emanating from Turkey [5,6]. The rarity of this condition in other races and regions is evidenced from the occasional reports from India [7-9] (Asia) and Sudan [10] (Africa). The report from Sudan was from the Arab North. There is no previous report in the English medical literature of cutaneous horns occurring in people from Sub-Saharan Africa. This may be the first such report involving a black African.

Bondeson presented an excellent review of cutaneous horns. In Europe these individuals were often treated with superstitious awe and many enterprising showmen made careers out of exhibiting people with cutaneous horns for money [1]. Yu et al reported a series of 643 patients over a 10 year period, with 32 new patients annually, while Mencıa-Gutierrez et al. presented 48 patients in Spain with eyelid cutaneous horns over a similar period of time [4]. Thus cutaneous horns may be considered a relatively common entity amongst Caucasian populations.

## Conclusion

Burn scars are known to heal with hypertrophic scars, keloid, skin dyspigmentation, and chronic non-healing or unstable scars which may degenerate into squamous cell carcinomas (Marjolin's ulcers), amongst other scar complications. However, there appears to be no previous mention in the literature of a cutaneous horn developing from a thermal burn scar.

The present case is reported so that cutaneous horns, a common entity in the West, but a rarity in black Africans, may be considered among the differential diagnosis in dermatological conditions. The rarity of this condition also lends itself to the most unusual interpretations and superstition, as evidenced by the curious interest and discussion in the operating room at the time of this particular patient's surgery, and it is hoped that this brief report will help to correct these misconceptions. Finally, up to 40% of cutaneous horns have been shown to have an underlying premalignant or malignant lesion, hence the importance of complete extirpation and histopathological diagnosis [3].

## Competing interests

The author(s) declare that they have no competing interests.

## Authors' contributions

The corresponding author came up with the idea, performed the write up and referencing. The author takes sole responsibility of the entire content of this article.

## Consent

Informed written consent was obtained from the patient for the publication of this paper.
